# A Self‐Powered Double U‐Finger MME Resonator Capable of Wirelessly Capturing Abnormal Message in Smart Grid Networks

**DOI:** 10.1002/advs.202508149

**Published:** 2025-07-11

**Authors:** Xinyi Zheng, Zhi Cheng, Bing Wang, Wei Peng, Yuelong Yu, Haoxian Peng, Yu Lei, Xiangmeng Lv, Jianglei Chang, Shitong Fang, Shuxiang Dong

**Affiliations:** ^1^ School of Mechatronics and Control Engineering Institute for Advanced Study Shenzhen University Guangdong 518060 China; ^2^ School of Materials Science and Engineering Wuhan University of Technology Wuhan 430070 China; ^3^ Key Laboratory of Inorganic Functional Materials and Devices Shanghai Institute of Ceramics Chinese Academy of Sciences Shanghai 201899 China; ^4^ Guangdong Key Laboratory of Electromagnetic Control and Intelligent Robots Shenzhen University Guangdong 518060 China; ^5^ School of Aerospace Engineering Beijing Institute of Technology Beijing 100081 China; ^6^ Electronic Materials Research Laboratory Key Laboratory of the Ministry of Education School of Electronic Science and Engineering Xi'an Jiaotong University Xi'an 710049 China; ^7^ School of Materials Science and Engineering Peking University Beijing 100871 China

**Keywords:** energy harvesting, magnetic sensing, magneto‐mechano‐electric coupling, self‐powered, smart grid monitoring

## Abstract

The magneto‐mechano‐electric (MME) coupling principle plays an important role in synchronously harvesting magnetic energy and monitoring safe operation in the distribution grid and the Power Internet of Things (PIoT). In this study, an MME resonator is presented, containing two crossed U‐fingers made of piezoelectric phase‐elastic beams with different lengths and magnet masses, operating in symmetric, decoupling dual bending modes. One U‐finger resonating at 50 Hz serves as energy harvester (EH), while the other resonating at 185 Hz acts as current or magnetic sensor, enabling the resonator to simultaneously and wirelessly capture 50 Hz stray magnetic field (*H_AC_*_,50 Hz_) energy and ground fault message of power lines by injecting an additional non‐grid‐frequency (185 Hz) current. The EH U‐finger generates an output power of 1.53 mW_RMS_ under a weak *H_AC_*_,50 Hz_ of only 0.5 Oe, achieving a normalized power density surpassing current standards. While the sensing U‐finger shows a high sensitivity to *H_AC_*_,185 Hz_ with a detectability of 710 pT, even the EH U‐finger is operating. The application test demonstrates the system's wireless monitoring and synchronous self‐powered functions, providing stable energy for self‐sensing data processing and transmission. This work introduces an efficient, wirelessly self‐powered, and self‐sensing method for advancements in PIoT.

## Graphical abstract

This study presents a magneto‐mechano‐electric resonator that efficiently harvests energy and monitors grid safety in Power Internet of Things systems. The resonator, consisting of two U‐fingers with different resonant frequencies, captures stray magnetic field energy and detects fault messages. It produces high power for self‐powered and achieves high sensitivity for wireless self‐sensing, offering a novel solution for autonomous grid monitoring.

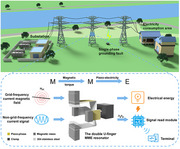

## 1 Introduction

Power Internet of Things (PIoT), as a vital technique in the smart grid, enabling seamless connectivity and autonomous monitoring across all stages of the power system.^[^ ^1^, ^2^ ^]^ PIoT is constituted by an amount of monitoring sensors, which are traditionally powered by batteries with limited capacity. When these batteries are depleted, interruptions in the monitoring system will result in the inability to promptly address potential hazards. Furthermore, the frequent replacements and maintenance of batteries lead to high economic and environmental costs. While the electric‐network‐based power supplying for large number, widely‐distributed sensor units is also unacceptably, because it means a large number of power‐interfaces and voltage transformations elements. In addition, they cannot meet the mobility needs. Thus, it remains a challenging task to provide sustainable energy sources for powering sensors to achieve real‐time monitoring of the distribution network operation status and fault detection.

To overcome the above challenges, many researchers proposed to capture solar energy,^[^ ^3^, ^4^ ^]^ thermal energy,^[^ ^5^ ^]^ vibration energy,^[^ ^6^, ^7^ ^]^ wind energy,^[^ ^8^ ^]^ and stray magnetic field energy^[^ ^9^, ^10^ ^]^ in the environment to power PIoT devices, and have made significant progress. Among them, the use of stray magnetic fields for energy harvesting （EH） in distribution networks shows unique advantages. Stray magnetic field is a form of stable and sustainable energy naturally generated during the operation of distribution grid. This EH method makes full use of the resource characteristics of the distribution grid itself, providing a reliable and sustainable solution for self‐powered sensor systems without the risk of disappearance.^[^ ^11^ ^]^ In order to utilize the stray magnetic field energy, the coils based electromagnetic induction have traditionally been used. However, within a limited volume, it is difficult to generate high output power under the low‐frequency magnetic field excitation.^[^ ^12^ ^]^ Thus, the magneto‐mechano‐electric (MME) energy harvester, which combines piezoelectric effect and magnetic torque effect, has been proposed to effectively convert magnetic field energy into electrical energy.^[^ ^13^ ^]^ In the early stage of the research, a typical MME energy harvester was composed of a cantilever beam composited with piezoelectric materials and attached with a permanent magnet at the free end, working in the first‐order bending mode.^[^ ^14^, ^15^ ^]^ Afterward, researchers further improved the performances of the MME energy harvester from both material and structure aspects. Annapureddy et al. used low‐loss PMN‐PZT SCMFs and Ni sheets to enhance the output performance of the energy harvester and achieved an output power of 0.73 mW under an excitation magnetic field of 7 Oe at 60 Hz.^[^ ^16^ ^]^ Subsequently, the output power was further improved by combining Fe‐Ga alloy with high magnetostrictive properties and piezoelectric single crystal fiber composites.^[^ ^17^ ^]^ As for the improvement from the structure aspect, Chu et al. proposed a clamped‐clamped MME energy harvester with higher loadable magnet mass, which achieved an output power of 0.97 mW under a weak magnetic field of 1 Oe at 50 Hz.^[^ ^18^ ^]^ Inspired by nature, Chang et al. introduced a dragonfly‐wing‐like MME energy harvester operating in two intercrossed symmetric bending modes and achieved an output power of 4.45 mW under a magnetic field of 1 Oe at 50 Hz.^[^ ^19^ ^]^ Finally, in order to achieve applications of MME energy harvesters in magnetic field environments, many researchers have applied them in self‐powered temperature and humidity sensing. For example, Yu et al.,^[^ ^20^ ^]^ Patil et al.,^[^ ^21^ ^]^ Yu et al.^[^ ^22^ ^]^ and other researchers have used MME energy harvesters to power temperature and humidity sensors and communication units, thus achieving continuous real‐time room temperature and humidity data measurement. Although researchers have made significant progress in the application of MME energy harvesters, they have not expanded it to other promising applications, and most MME devices require external sensors to realize self‐powered sensing applications without self‐sensing functions.

In the distribution grid, after a single‐phase grounding fault occurs in a medium or low voltage network, the system can continue operating for a period of time since the line voltage remains unchanged. However, if the fault is not correctly identified and removed in time, it may cause greater damage to the distribution network.^[^ ^23^, ^24^ ^]^ Therefore, the rapid identification and removal of single‐phase ground faults plays an important role in ensuring the safe operation of distribution networks. The current methods for detecting single‐phase ground faults include impedance method,^[^ ^25^ ^]^ traveling wave method,^[^ ^26^ ^]^ and signal injection method.^[^ ^27^ ^]^ Signal injection methods only require current measurements along the distribution feeder and thus have been widely used in single‐phase ground fault detection.^[^ ^28^ ^]^ In order to avoid increasing the fault current, variable‐frequency current injection method is often used. For instance, Wang and Zhang injected 220 Hz sinusoidal AC signal into the distribution network to locate ground faults.^[^ ^29^ ^]^ Traditionally, the sensing devices for detecting the variable‐frequency current include fluxgate sensors,^[^ ^30^ ^]^ current transformers,^[^ ^31^ ^]^ hall current sensors.^[^ ^32^ ^]^ However, these current sensors have the disadvantages of weak signals, and low sensitivity. Compared with these, the MME sensors mainly operate at a certain resonant frequency, and can effectively generate the high magnetoelectric (ME) voltage under the magnetic field generated by the variable‐frequency current signal for sensing the current.^[^ ^33^, ^34^ ^]^ Furthermore, the MME devices are small in size, light in weight, and has a simple structure with ease of integration in the distribution network. However, although the MME sensors can complete the function of signal acquisition, subsequent functions such as signal reading and data transmission still need external power supplies, which limits these devices in scenarios with high requirements in energy independence and long‐term uninterrupted operation. In contrast, MME devices with synchronous self‐powered and self‐sensing functions can provide stable energy support for the self‐sensing data processing and transmission, thus providing a more efficient and sustainable solution for the ground fault detection. Although researchers have made significant progress in the self‐powered and self‐sensing MME devices, most studies can only achieve a single function, or cannot achieve dual functions at the same time. This is mainly due to the fact that these structures can only operate at a single frequency of magnetic field. Chang et al. proposed a self‐powered MME sensor with a dragonfly wing structure, which achieved an output power of 1.24 mW and a magnetic field detection capability of 2.7 nT at 60 Hz and 1 Oe.^[^ ^35^ ^]^ Gerhardt et al. designed a cantilever‐beam structured self‐powered hybrid magnetoelectric sensor, achieving a detection limit of 46 pT/√Hz and a power density of 1.31 µW cm^−3^ Oe^−2^ at 223.5 Hz, respectively.^[^ ^36^ ^]^ For these MME devices, the energy harvesting and sensing functions cannot be simultaneously achieved.

**Figure 1:**
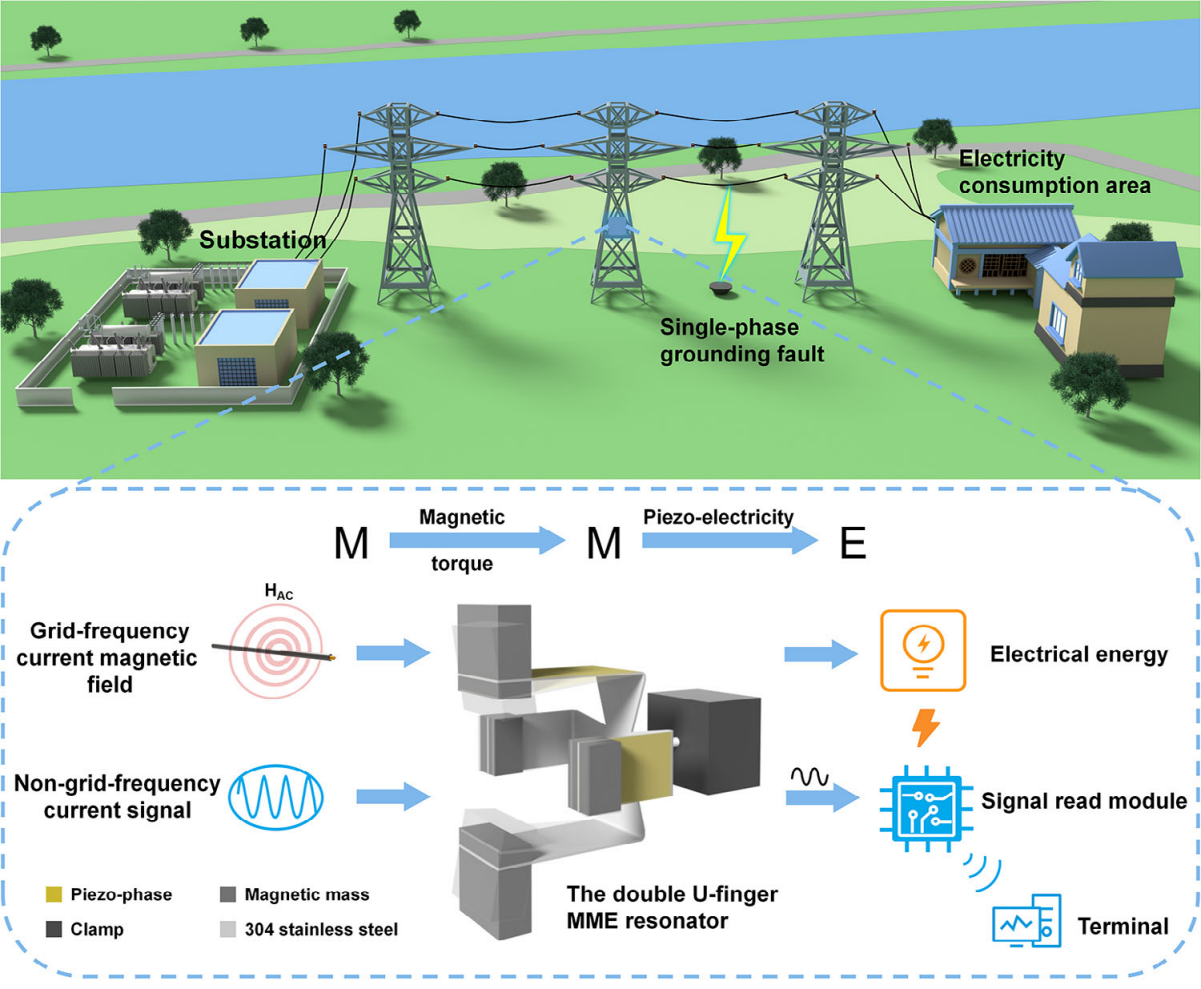
The working principle of a double U‐finger MME resonator and schematic illustration of the operation in a distribution network environment.

To address this situation, we propose a double U‐finger MME resonator, like men's fingers, simultaneously capturing energy and weak abnormal‐message through MME coupling in distribution grid. The application scenario and working principle of double U‐finger MME resonator are shown in **Figure** **1**. The resonator integrates two U‐shaped beam structures perpendicular to each other with unequal lengths, which can simultaneously realize the functions of self‐powered and self‐sensing under the same‐direction incident magnetic field. Furthermore, at a resonant frequency of 50 Hz, the proposed resonator is demonstrated to achieve an output power of 1.53 mW_RMS_ under a magnetic field of 0.5 Oe, while under an extremely weak magnetic field of 0.1 Oe, its normalized power density reaches 10.97 µW_RMS_ Hz^−1^ Oe^−2^ cm^−3^. This marks a 12.7% improvement in normalized power density over existing highest‐performing MME‐EHs.^[^ ^19^ ^]^ Besides, it demonstrates a high magnetic field detection capability of 710 pT at 185 Hz with neglectable interference from the EH U‐finger. As a practical application, by capturing the stray magnetic field energy around the power line, the signal reading module, and the communication module can be powered to transmit the ME voltage signal captured by the sensing U‐finger, thus reflecting the magnitude of the non‐grid‐frequency current. The proposed synchronous self‐powered and self‐sensing MME resonator demonstrates high application potential in self‐powered ground fault detection, which is of significance for the development of PIoT.

## 2 Results and Discussion

### 2.1 Design of the Double U‐Finger MME Resonator

The proposed double U‐finger MME resonator consists of two crossed U‐shaped piezoelectric beams A and B, as EH U‐finger and sensing U‐finger, respectively, perpendicular to each other with different lengths. Each U‐finger in this resonator contains one pair of symmetric beams made of elastic metal beam composited with one piezoelectric ceramic sheet, and each pair are attached with one pair of symmetrically arranged permanent magnets at the ends. Under the certain excitation magnetic field, each pair of beams vibrates symmetrically, forming decoupling dual‐mode bending vibrations due to different resonance frequency, depending on their geometric shapes and dimensions. Through the careful geometry design, the required resonance frequency for each pair of U‐fingers can be achieved, allowing this MME resonator to respond variable‐frequency magnetic fields. In addition, the resonant frequencies of the two U‐fingers are different, with a significant difference. Therefore, each U‐finger in this MME resonator works independently and their output signals almost do not affect each other due to decoupling effect.

**Figure 2:**
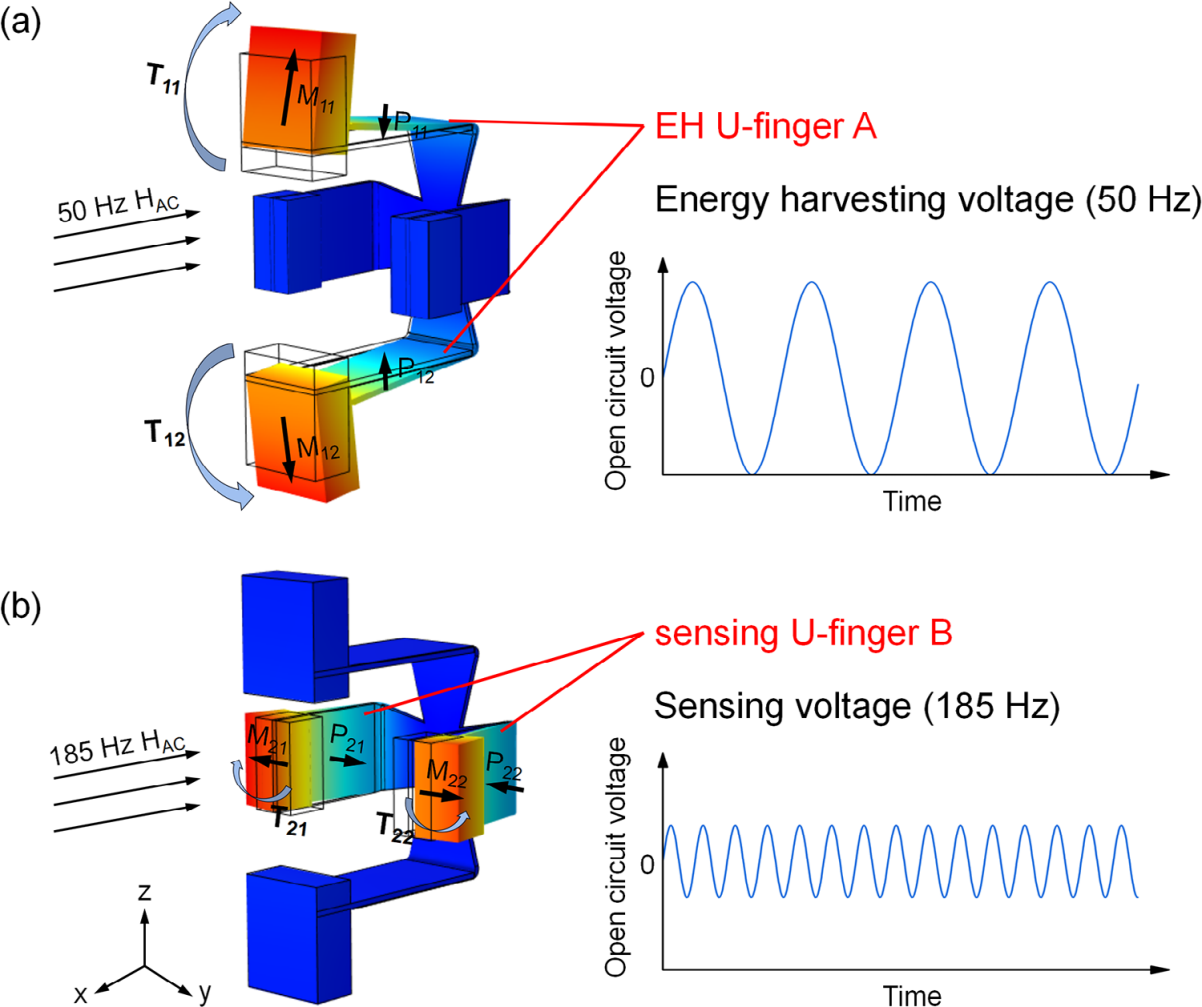
a,b) Schematic view of the symmetrical, decoupling dual‐mode bending vibrations of the double U‐finger MME resonator at 50 and 185 Hz, respectively.

**Figure** **2**a,b shows the two vibration modes of the resonator, including the magnetization directions of the magnets, and the polarization directions of the piezoelectric sheets. We make the magnetization and polarization directions of the magnets and piezoelectric sheets on both sides of U‐finger A and U‐finger B opposite to each other, so that they can work in a symmetric bending mode. At the same time, by arranging two U‐fingers vertically, the resonator can have two frequency vibration modes with almost no interference between them. More specifically, one pair of U‐finger A is designed to be suitable for efficiently harvesting the stray magnetic field energy generated by the grid frequency current of the distribution network, achieving self‐power supply function; while the designed U‐finger B has the ability to sense the non‐grid‐frequency injection current through the magnetic field signals generated around the current. Such dual‐frequency characteristic of MME resonators realizes the multiple functional synergy: i) power harvesting from grid‐frequency current, and ii) signal sensing via non‐grid‐frequency current injection for ground fault detection, and iii) self‐powered signal transmission.

At the same time, in order to increase the ability of MME coupling, thus obtaining better energy harvesting and sensing abilities, each pair of U‐fingers is designed with variable stiffness and dual‐mode vibration characteristics. Under the magnetic field excitation, the stress concentration due to the variable stiffness design of the substrate near the clamping end results in larger beam vibration amplitudes. In addition, by adjusting the distance between the two arms, the amplitude at the end of the piezoelectric pieces can be increased. The mirror structure design of the two U‐shaped beams enables the resonator to operate in mechanical coupled symmetric dual‐mode vibrations. Therefore, the clamping part is almost stationary during the vibration process due to the formation of a natural node line, which greatly reduces the clamping loss and improves the mechanical quality factor. At the same time, the symmetrical, decoupling dual‐mode vibrations will undergo strong MME coupling, thus improving the energy conversion efficiency. In summary, the proposed resonator can realize the functions of high‐performance self‐powered and self‐sensing simultaneously, showing great potential in building a more intelligent and sustainable PIoT.

### 2.2 MME Coupling Analysis

In the research field of MME energy harvesters and sensors, increasing ME output voltage and optimizing power output are core goals, and magneto‐mechanical coupling and electromechanical coupling are the key mechanisms to achieve these goals.^[^ ^37^ ^]^ Finite element analysis (FEA), mechanical analysis, and experimental analysis are used in this section to deeply explore the coupling process in the double U‐finger MME resonator.

**Figure 3:**
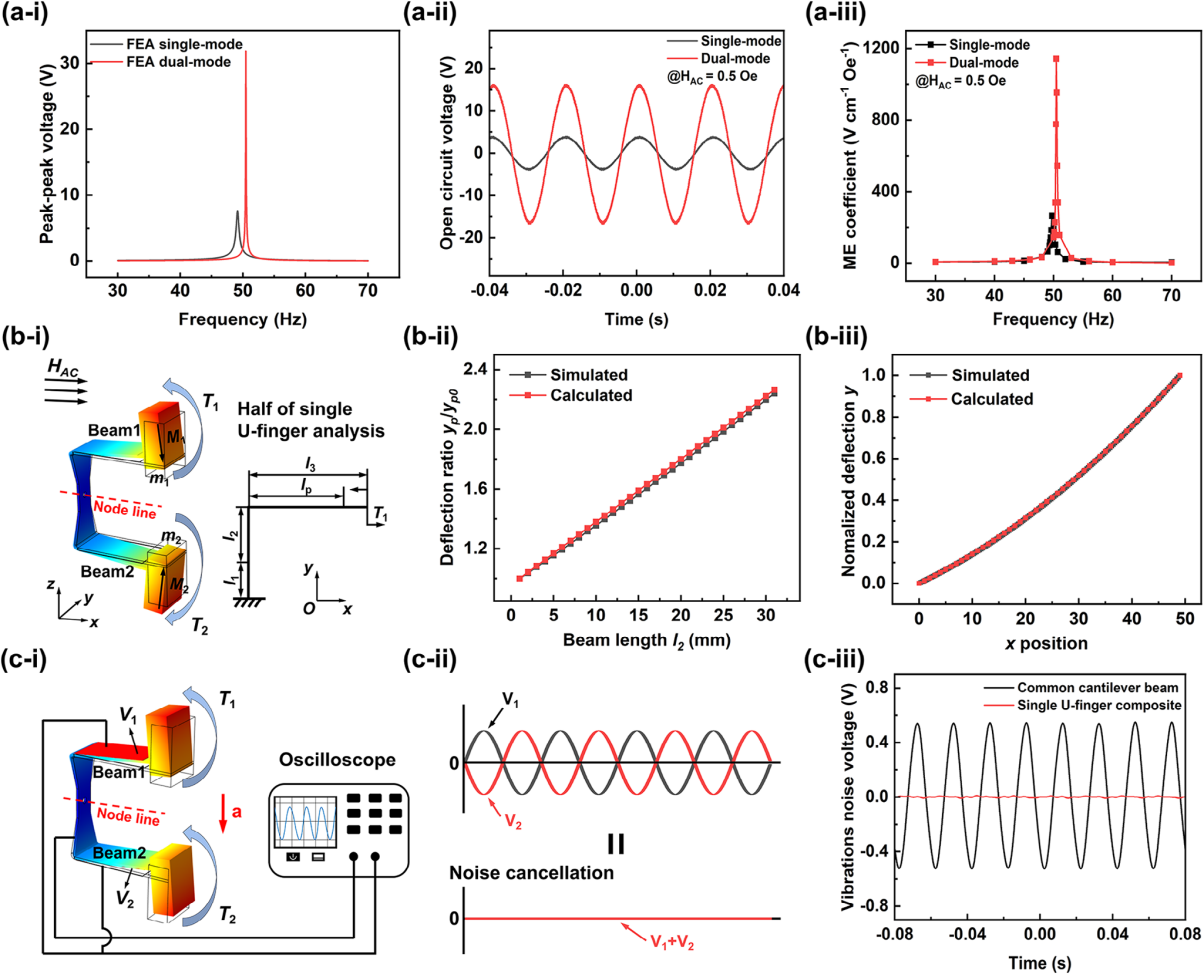
MME coupling analyses and measurements of the double U‐finger MME resonator. Simulated frequency‐domain (a‐i) and measured time‐domain (a‐ii) peak‐peak output voltages of the single U‐finger resonator, when operating in dual‐mode and single‐mode vibrations, respectively. (a‐iii) Measured magnetoelectric coupling coefficient in frequency‐domain for single‐mode and dual‐mode vibrations, respectively. b‐i) Schematic view of the vibration mode of the U‐finger under *H_AC_* and its dimensional illustration. b‐ii) The calculated and simulated piezoelectric end displacement ratio y_p_/y_p0_ varies with the beam length *l_2_*. b‐iii) The calculated and simulated the normalized deflection *y* of Beam 1 as a function of *x*. c‐i) Schematic diagram of the vibration mode of the U‐finger under external vibration acceleration *a* (noise) excitation. c‐ii) Schematic diagram of the cancellation mechanism of vibration noise. c‐iii) Restrained noise signal (red line) under external vibrations (acceleration *a* = 0.06 g) in U‐finger.

The double U‐finger MME resonator can be decomposed into two mutually perpendicular U‐fingers; while two arms in each U‐finger operate in symmetric bending mode under the incident AC magnetic field H_AC_. Therefore, the following analysis will be firstly based on one single U‐finger resonator. Since the two arms of one single U‐finger resonator are symmetrical structure, they couple in a symmetric dual‐mode vibrations under H_AC_ excitation. In this case, the clamping part is located on the natural node line, and it remains an almost static state during vibrations, which effectively restrains the clamping energy loss and therefore improves the electromechanical coupling effect. When one arm of the U‐finger resonator is fixed, the structure becomes an L‐shaped beam, i.e., a traditional single‐mode vibration beam. In order to compare the effect of clamping loss between the dual‐mode symmetric vibration structure and the single‐mode vibration structure, finite element analysis (FEA) is used to simulate their output voltage responses. **Figure** **3**a‐i shows the peak‐peak output voltage of the U‐finger in symmetric vibration mode and that of the L‐shaped beam in single‐vibration mode in time‐domain, respectively. It can be found that at the resonant frequency, the peak‐peak output voltage of the L‐shaped beam is only 7.65 V_pp_, while the peak‐peak output voltage of the U‐finger is as high as 32.17 Vpp, which is 4.2 times that of the L‐shaped beam. In addition, we calculated their mechanical quality factor Q_m_ through measuring resonant frequency *f_r_* and 3 dB bandwidth *∆f*, and the estimated Q_m_ for the U‐finger and the L‐shaped beam are 297 and 124, respectively. These results indicate that dual‐mode symmetrical vibrations of the U‐finger can effectively suppress clamping energy loss and improve the mechanical quality factor, thereby significantly enhancing the energy conversion efficiency and sensitivity of the U‐finger.

In addition, we also conducted the analysis through experiments. To work at the first‐order bending resonant frequency of 50 Hz, we symmetrically configure one pair of permanent magnets (*m_1_* and *m_2_*) with reversed magnetization but with the same volume (mass) and their positions on both Beams 1 and 2, so that the U‐finger resonator produces symmetrical dual‐mode vibrations at exact 50 Hz. When fixing one arm (Beam 2 with *m_2_* = 0), then the U‐finger resonator produces single‐mode bending vibrations. Figure 3a‐ii shows the output voltage waveforms of the U‐finger resonator in time‐domain when operating in single‐mode and dual‐mode bending vibrations, respectively. Again, it can be found that when the U‐finger works in single‐mode, the output voltage (from Beam 1) is only 7.6 V_pp_. When the U‐finger works in dual‐mode, output voltages of its each beam are significantly increased to 32.2 V_pp_. Besides, Figure 3a‐iii also shows the comparison on the magnetoelectric coupling coefficients *α_ME_* in frequency domain when operating in single‐mode and dual‐mode, respectively, under H_AC_ of 0.5 Oe. It is seen that *α_ME_* for single‐mode MME coupling is only 263.6 V cm^−1^ Oe^−1^, while *α_ME_* for dual‐mode MME coupling is as high as 1142.4 V cm^−1^ Oe^−1^, which is 430% higher than that operating in single‐mode MME coupling. Such an improvement in the magnetoelectric coupling coefficient is not only helpful to improve the energy conversion efficiency when used as energy harvester, but also helpful to enhance the sensitivity and detection accuracy of the U‐finger MME resonator when used as a sensor.

In addition, the mechanical analysis is used to analyze the relationship between the bending displacement at the end of the piezoelectric piece attached on the arms and the distance between the two arms under H_AC_. Since the U‐finger structure is symmetrical, only half of the beam is analyzed. The distance between the two arms is adjusted through the length of l_2_, as shown in Figure 3b‐i. Since the contribution of the piezoelectric phase to the bending stiffness of the U‐finger is very small, in order to simplify the mechanical analysis, the size effect of the piezoelectric phase is ignored. Therefore, the horizontal displacement and the vertical displacement of the arm in the U‐finger can be respectively expressed as:

(1) $$\begin{array}{cc} x & =\frac{C}{A^{2}}\left(\left(Al_{2}+B\right)\ln\left(Al_{2}+B\right)-Al_{2}+B\right)+\left(\theta_{b}-\frac{C}{A}\ln B\right)l_{2}+w_{b}\\ & \;-\frac{C}{A^{2}}\left(B\ln B-B\right) \end{array}$$

(2) $$y=\left(\frac{C}{A}\ln\left(Al_{2}+B\right)+\theta_{b}-\frac{C}{A}\ln B\right)x+\frac{6T_{1}x^{2}}{Ea_{2}b^{3}}$$

where A = E(a_2_‐a_1_)b^3^, B = Ea_1_b^3^l_2_, C = 12T_1_l_2_. E is the Young's modulus of the beam, l_1_ is the length of the longitudinal beam with constant stiffness. l_2_ is the length of the longitudinal beam section with variable stiffness. l_3_ is the length of the transverse beam section. a_1_ is the width of the longitudinal beam with constant stiffness. a_2_ is the width of the transverse beam section. θ*_b_* and *w_b_* are the rotation angle and deflection of the connection part between the longitudinal beam and the transverse beam sections, respectively. When the length of the piezoelectric piece is fixed at l_p_, the vertical displacement at the piezoelectric piece end can be expressed as:

(3) $$y_{p}=\left(\frac{C}{A}\ln\left(Al_{2}+B\right)+\theta_{b}-\frac{C}{A}\ln B\right)l_{p}+\frac{6T_{1}l_{p}^{2}}{Ea_{2}b^{3}}$$

Under the condition of fixing total beam length, the relationship between the end displacement of the piezoelectric piece and the distance between the two arms (parameter *l_2_*) can be calculated by using theoretical analysis and FEA, respectively. The displacement at the end of the piezoelectric piece when *l_2_* = 0 mm is selected as the reference value y_p0_. As l_2_ increases, the ratio of the displacement y_p_ at the end of the piezoelectric piece to the reference value y_p0_ is shown in Figure 3b‐ii. It can be seen that the theoretical analysis and FEA results are basically consistent. The results show that under the same magnetic torque, the larger the distance between the two arms, the greater the end displacement of the piezoelectric piece, thus generating higher electrical energy. In addition, as shown in Figure 3b‐iii, the beam deflection *y* at any point *x* along the horizontal direction calculated by FEA and mechanical analysis is basically consistent, proving the accuracy of the calculation results. The detailed derivation and formula are given in Section S2 (Supporting Information).

Finally, it should be pointed out that the proposed U‐finger operates in the anti‐phase vibration mode under incident AC magnetic field H_AC_; but under the action of external environmental vibration, it will operate in the same direction vibration mode. As shown in Figure 3c‐i, the voltage generated by Beam 1 is V_1_, and the voltage generated by Beam 2 is V_2_. Since the polarization directions of the piezoelectric ceramics on the two arms are opposite and are connected in parallel at the same time, when subjected to external vibration, an output signal with opposite amplitude will be generated to offset the vibration noise, as shown in Figure 3c‐i. We conducted tests on the U‐finger and measured the signal output caused by vibration noise at an acceleration of ≈0.06 g. As shown in Figure 3c‐iii, the output voltage of Beam 1 is measured to be 0.371 V_RMS_ firstly, which is similar to the vibration noise of a traditional single cantilever beam. Then after beam 1 and beam 2 are connected in parallel, the vibration noise is significantly reduced to only 0.004 V_RMS_. This vibration suppression property is crucial in sensors, as it can effectively improve measurement accuracy and stability and enhance its adaptability in harsh environments.

Therefore, based on these theoretical analyses and measurements, it can be predicted that the proposed double U‐finger MME resonator will produce both superior energy harvesting and sensing performances in the weak AC magnetic field circumstance.

### 2.3 Energy Harvesting and Sensing Performances

**Figure 4:**
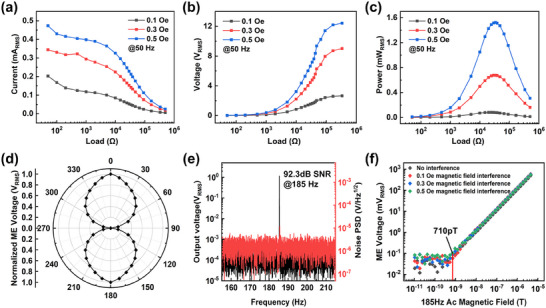
Energy harvesting and magnetic field sensitivity performances of the double U‐finger MME resonator. a–c) Output RMS voltage, current, and power as a function of load resistances under varying H_AC,50 Hz_. d) ME output voltage response when the sensing U‐finger rotates about the *z*‐axis. e) ME output voltage of the sensing U‐finger in response 185 Hz, 10 µT AC magnetic field, and noise power spectrum density (N_PSD_) in the absence of incident magnetic field excitation. f) the magnetic‐field sensitivity and linear response to AC magnetic field varying from 10 pT to 4 µT at the resonance frequency of 185 Hz.

To investigate the energy harvesting (EH) capability in stray magnetic fields, the EH U‐finger in the double U‐finger MME resonator is set to work in dual (first‐order) bending vibration mode with the resonant frequency of 50 Hz, and its output performances under different incident magnetic field strengths at 50 Hz are investigated systematically. The measurement circuit diagram is shown in Figure S3 (Supporting Information). The double U‐finger MME resonator is placed in the center area of one Helmholtz coil. After energizing the Helmholtz coil, an oscilloscope is used to display the output voltage waveform generated by the EH U‐finger in real‐time, as shown in Figure S4 (Supporting Information). At the same time, one resistance box is connected with the EH U‐finger in series for measuring the output current, output voltage, and output power, respectively, as shown in **Figure** **4**a–c. Under the incident magnetic field of H_AC_ = 0.1, 0.3, and 0.5 Oe, the corresponding maximum output powers are 0.08, 0.68, and 1.53 mW_RMS_, respectively, at the load resistance around 30 kΩ. It is found that the maximum output power up to 4 mW_RMS_ can be generated when H_AC_ increases to 1 Oe. Figure S5 (Supporting Information) shows the output power of the EH U‐finger A as a function of incident magnetic field H_AC_ in the range of 0 ~1 Oe. In addition, in order to study the effect of the incident magnetic field direction on the output power of the EH U‐finger A, the double U‐finger MME resonator is rotated around the z‐axis as shown in Figure S6a (Supporting Information), and the corresponding maximum output power at different direction angles is measured. Figure S6b (Supporting Information) shows the polar coordinate diagram of the normalized output power corresponding to different incident magnetic field direction. It can be observed that when the double U‐finger MME resonator is oriented toward (180 ) or along (0 ) the incident magnetic field H_AC_ direction (along the x‐axis), the output power reaches the maximum value; in contrast, when the double U‐finger MME resonator is perpendicular to the H_AC_ direction (along the y‐axis), its output power decreases to the minimum value. Finally, Figure S7 (Supporting Information) shows that the EH U‐finger still exhibits a stable voltage response after operations lasting over 5.7  × 10^6^ cycles under the excitation of an AC magnetic field of 0.3 Oe with the resonant frequency of 50 Hz. This fatigue test validates the reliability of the double U‐finger MME resonator under long‐term operation.

**Table** **1** shows the comparison of the output performance of the EH U‐finger with those of other reported MME‐EHs operating in the industrial frequencies. It can be found that compared with previously reported MME‐EH using piezoelectric ceramics, the proposed EH U‐finger exhibits a higher output power density. Specifically, under the excitation magnetic field of 0.5 and 0.1 Oe, the output power density of the proposed double U‐finger MME resonator can reach 0.41 mW_RMS_ cm^−3^ Oe^−2^ and 0.55 mW_RMS_ cm^−3^ Oe^−2^, respectively, which show an increment up to 104% compared with the current best MME‐EH using piezoelectric ceramics.^[^ ^20^ ^]^ Notably, this EH U‐finger using cheaper piezoelectric material of PZT‐5H ceramic obtains a normalized power density per Hz of 10.97 µW_RMS_ Hz^−1^ Oe^−2^ cm^−3^ at an excitation magnetic field of 0.1 Oe, which is even superior to that of MME‐EH using more expensive single crystal, indicating an enhancement by 12.7% over the state‐of‐art methods.^[^ ^9^, ^38^, ^39^, ^40^ ^]^

**Table 1:** Comparison of the magnetic field energy harvesting performances the double U‐finger MME resonator and previously reported MME‐EHs operating in low‐frequency range of 50/60 Hz.

Piezoelectric materials	Year	Structure	ME composite	Working frequency (Hz)	Excitation field (Oe)	Output power [mW_RMS_]	Normalized power [mW_RMS_ Oe^−2^]	Normalized power density [mW_RMS_ Oe^−2^ cm^−3^]	Normalized power density per Hz [µW_RMS_ Hz^−1^ Oe^−2^ cm^−3^]	References.
Ceramics	2025	EH U‐finger	PZT‐5H/304	50	0.1	0.08	8.23	0.55	10.97	This work
					0.5	1.34	5.37	0.41	8.26	
	2024	I‐Shaped MME	PZT‐5H/ST	60	0.75	7.31	13	0.18	3	[22]
	2024	T‐Shaped MME	PZT‐5H/ST	60	1.75	12.3	4.02	0.27	4.5	[20]
	2023	Dragonfly‐like wing	PZT‐5H/TC4	50.5	1	4.45	4.45	0.26	5.15	[19]
	2022	Tuning fork structured MME	PZT‐5H/ST	60	1	≈1.1	≈1.1	0.16	2.73	[41]
	2022	Clamped‐Clamped MME	PZT‐5H/copper	50	0.48	0.37	1.61	0.15	2.96	[18]
	2020	Cantilevered MME	PZT‐5A/ Metglas	60	0.5	0.17	0.68	0.24	4	[42]
Single crystal	2023	Cantilevered MME	PMN‐PZT SFC/Fe‐Ga	60	3	7.4	0.82	0.19	3.17	[38]
	2023	Cantilevered MME+ flux concentrator	PMN‐PZ‐PT SFC/Ni	60	1	0.25	0.25	0.58	9.73	[39]
	2021	Cantilevered MME+ dual mass	PMN‐PZT SCMF/Metglas	60	4	1.25	0.08	0.06	1	[40]
	2020	Cantilevered MME+ flux concentrator	Mn‐doped PMN‐PZT SCMF/Ni	60	8	3.3	0.05	0.08	1.33	[9]

In fact, as mentioned in Section 2.1, the double U‐finger MME resonator consists of two crossed U‐fingers, one for magnetic field energy harvesting, and another for current or magnetic field sensing. As we have seen above, the EH U‐finger in the MME resonator has shown the great advantages in magnetic field energy harvesting applications. We will see that the sensing U‐finger in the resonator also shows superior magnetic sensing performance. It is because the symmetrical first‐order resonant bending mode of the sensing U‐finger would produce a restrain effect to environment vibration noise. In addition, compared with other magnetoelectric sensors, this double U‐finger MME resonator does not require an additional bias magnetic field, and the readout technology is also simple because the voltage signal strength generated by the sensing U‐finger is strong enough to be detected.

To investigate the directional sensing of incident magnetic field, a Helmholtz coil is used to generate H_AC_, and the double U‐finger MME resonator is rotated about z‐axis inside the coil for testing the optimal direction response, as shown in Figure S6a (Supporting Information). The normalized output ME voltage responding to different incident angles of H_AC_ exhibits a polar plot, as shown in Figure 4d. It can be observed that the ME voltage reaches the maximum value when the double U‐finger MME resonator is toward (180 ) or along (0 ) the incident magnetic field H_AC_ direction (along *x*‐axis), while it reaches the minimum value when the double U‐finger MME resonator is vertical to H_AC_ direction (along the y‐axis). Therefore, in the following test, the double U‐finger MME resonator is arranged along the *x*‐axis direction for detecting the incident H_AC_. Correspondingly, Figure 4e shows the output ME voltage of the sensing U‐finger in response to an excitation AC magnetic field of H_AC_ = 10 µT at a frequency of 185 Hz, and the voltage noise spectrum in the frequency range of 155 to 215 Hz. It can be concluded that the sensing U‐finger responds to the AC magnetic field with an output of 1.17 V, and the noise power spectrum density at this frequency is only 2.08 µV/√Hz.

To be closer to actual working conditions, we tested the detection limit of the sensing U‐finger while allowing the EH U‐finger to work at grid frequency of 50 Hz under different magnetic field strengths. For the detection limit analysis of the sensing U‐finger, a lock‐in amplifier is used to excite the coil for generating an AC magnetic field and detect the ME voltage generated by the sensing U‐finger under this magnetic field at the same time. Figure 4f shows the output voltage of the sensing U‐finger in response to a 185 Hz AC magnetic field (*H_AC_*_,185 Hz_) while the resonator is synchronously harvesting the energy of 50 Hz AC magnetic field (*H_AC_*_,50 Hz_) varying from 0.1 to 0.5 Oe. The measured ME voltage of the sensing U‐finger exhibits a clear linear response to the 185 Hz incident magnetic field. It can be found that the sensing U‐finger is only sensitive to H_AC,185 Hz_ even when the sensing U‐finger and the EH U‐finger are performed simultaneously. The two crossed U‐fingers are hardly affected by each other due to the decoupling effect between them. From Figure 4f, the calculated sensitivity (S_m_) at the sensing frequency of 185 Hz is in the range of 101.02–102.68 kV T^−1^, with a fluctuation of only 1.66 kV T^−1^, and the limit of detection (LOD) is 710 pT. This fully demonstrates the robustness of the magnetic field detection of the double U‐finger MME resonator in the presence of different strengths of the non‐grid‐frequency magnetic field interference. The equivalent magnetic noise (N_m_) can be calculated as follows:

(4) $$N_{m}=\frac{N_{\mathrm{PSD}}}{S_{m}}=\frac{\left(2.08\mu V/\sqrt{\mathrm{Hz}}\right)}{\left(1.02\times 10^{5}V/T\right)}=2.04\frac{\mathrm{pT}}{\sqrt{\mathrm{Hz}}}$$

In order to demonstrate the stability of the sensing performance of the double U‐finger MME resonator, a lock‐in amplifier is used to monitor its real‐time ME voltage response under varying AC magnetic field. Figure S8 (Supporting Information) shows that the double U‐finger MME resonator has an accurate step response to changes in the AC magnetic field (5 nT – 10 µT).

### 2.4 Synchronous, Wireless Self‐Powered, Self‐Sensing and Self‐Communication Functions

As mentioned before, rapid identification and removal of single‐phase ground faults play an important role in ensuring the safe operation of distribution networks. When a single‐phase grounding fault occurs in a low‐current grounding system, the non‐grid‐frequency current injection method may be used to locate the fault. In this case, the power line contains two currents with different frequencies. To enable this device to synchronously achieve magnetic field energy harvest function at grid‐frequency and injection‐current detection function at non‐grid‐frequency, a MME resonator with two crossed U‐shaped magnetoelectric fingers, EH U‐finger A and sensing U‐finger B, is designed. The two U‐fingers are arranged perpendicular to each other and have a common substrate at the center. Furthermore, the lengths and the attached magnet mass of two U‐fingers are carefully designed, making the two U‐fingers have different first‐order bending resonant frequencies. More specifically, the resonant frequency of EH U‐finger A is adjusted to be 50 Hz, corresponding to the power frequency. Whereas the resonant frequency of sensing U‐finger B is adjusted to be 185 Hz, corresponding to the non‐grid‐frequency of injection current.

**Figure 5:**
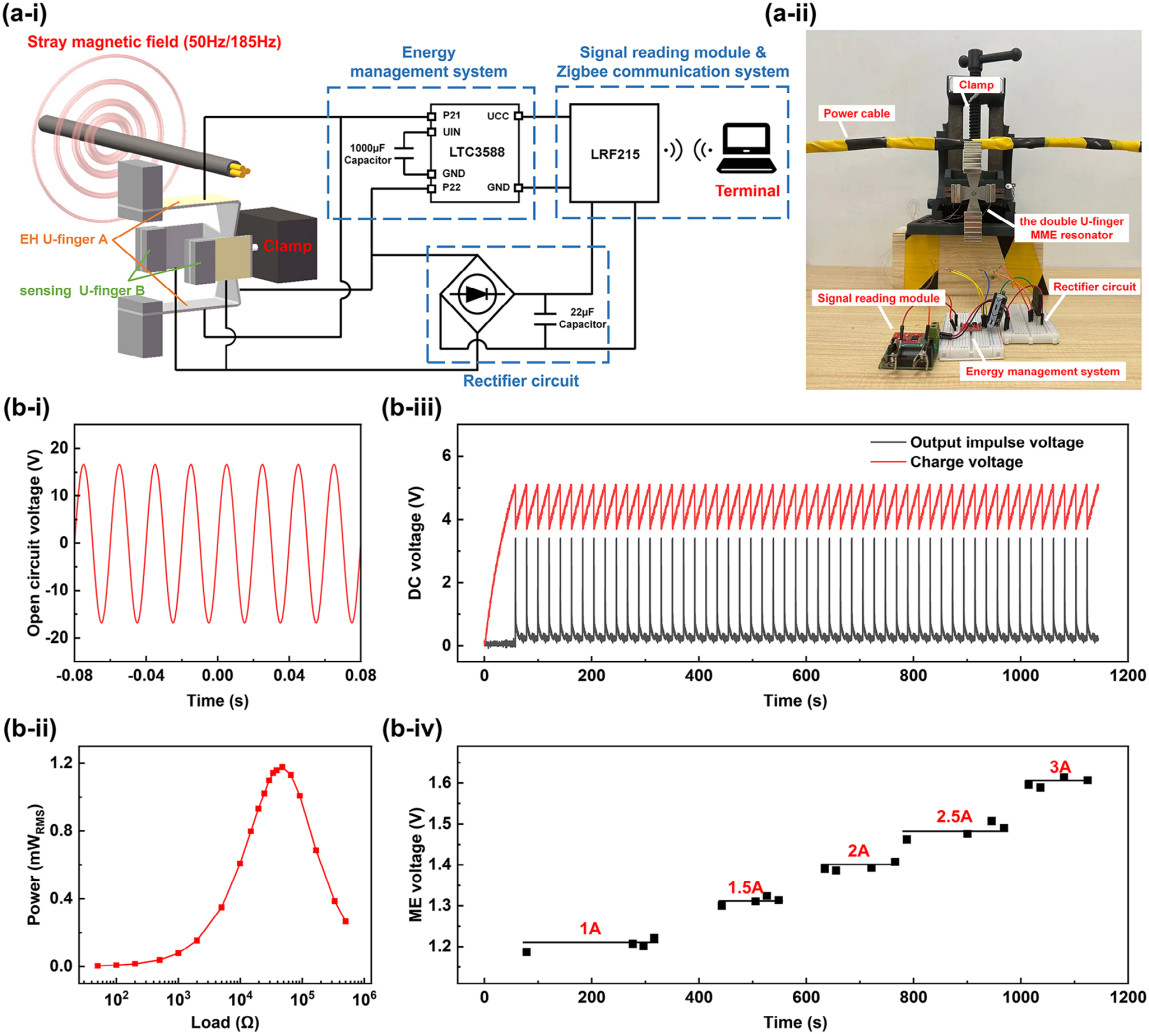
The double U‐finger MME resonator for the application for ground fault detection and data transmission. a‐i, ii) Working schematic of the double U‐finger MME resonator and photograph of the test setup. b‐i, ii) The voltage waveform and output power generated by EH U‐finger A near grid‐power line. b‐iii) The charging voltage and output impulse voltage curves of the energy management system when connecting to the signal reading module and communication system. b‐iv) The recorded ME voltage information is transmitted from the signal reading module to the terminal by the communication system.

As shown in **Figure** **5**a‐i, ii, a self‐powered non‐grid‐frequency current monitoring and communication system is built to evaluate the feasibility of the synchronous self‐powered and self‐sensing double U‐finger resonator for PIoT application. The system includes an EH U‐finger, a sensing U‐finger, an energy management system, a rectifier circuit, a signal reading module and a Zigbee wireless communication module. First, we inject 50 and 185 Hz AC currents into the power line, respectively, where the 50 Hz AC current is 5 A. Then, the EH U‐finger A converts the magnetic field energy around the power line of 50 Hz AC current into AC voltage (electrical energy) and then inputs it into the energy management system. The energy management system integrates the LTC3588 chip and the 1000 µF capacitor, which rectifies the input AC voltage into DC voltage and synchronously outputs DC voltage pulses continuously. The output DC voltage pulses power the signal reading module LRF215 and the Zigbee wireless communication module. At the same time, the rectifier converts the voltage response of the sensing U‐finger B to the *H_AC_*_,185 Hz_ around the power line into a DC voltage and stores it in a 22 µF capacitor. While the signal reading module LRF215 reads the DC voltage, after which the data is sent to the signal‐receiving terminal through the Zigbee wireless communication system. Finally, the received data corresponding to non‐grid‐frequency current can be treated there and judged if a grounding fault occurred.

The variation of the non‐grid‐frequency current in power lines is detected using the proposed self‐powered non‐grid‐frequency current monitoring and communication system. Figure 5b‐i shows the output voltage waveform of EH U‐finger A of the MME resonator under *H_AC_*_,50 Hz_ generated by 5 A alternating current, which reaches 47.4 V_pp_. Correspondingly, it generates a maximum output power of 1.18 mW_RMS_ at the load resistance of ≈30 kΩ (Figure 5b‐ii). As shown in Figure 5b‐iii, during the initial charging process, the voltage of energy management system reaches 5.1 V in 57.3 s, and then discharges, generating output impulse voltage with an amplitude of 3.37 V, after which the voltage of energy management system drops from 5.1 to 3.68 V within 0.6 s. After finishing first time discharge, the voltage of energy management system restores to its initial value within only ≈21 s because of continually charging the storage capacitor by the EH U‐finger A. Figure 5b‐iv shows the ME voltage data received by the terminal when the non‐grid‐frequency current size varies from 1 to 3 A with an interval of 0.5 A. Correspondingly, the magnitude of the non‐grid frequency current in the power line and the corresponding ME voltage data are shown in Figure S9 (Supporting Information). After linear fitting, the determination coefficient R^2^ is 0.99. It can be seen that the ME voltage data and the non‐grid‐frequency current size received by the terminal exhibit a linear relationship. A large ME voltage means a high non‐grid‐frequency injection current, and once the ME voltage is exceeding a threshold value V_T_, indicating a grounding fault occurring. It is worth noting that since LRF215 does not have a frequency selection function, the measured voltage will include the bias voltage caused by the EH U‐finger A. The above application tests demonstrate the potential of the proposed synchronous, wireless self‐powered, and self‐sensing MME resonator for achieving the self‐powered ground fault detection function in PIoT.

## 3 Conclusion

In summary, this work reports an innovative magneto‐mechano‐electric (MME) coupling resonator consisting of two crossed, piezoelectric phase‐elastic sheet composited U‐fingers, U‐finger A for capturing energy and U‐finger B for capturing weak abnormal‐message, attached with permanent magnetic mass pairs, and operating in symmetrical, first‐order dual‐mode decoupling bending vibrations. This double U‐finger MME resonator can simultaneously realize wirelessly self‐powered and self‐sensing functions, in which the EH U‐finger A is designed to be longer with a resonant frequency of 50 Hz, being responsible for harvesting magnetic field energy of grid‐power line; while U‐finger B is designed to be shorter with a resonant frequency of 185 Hz, being responsible for detecting the non‐grid‐frequency injection current for grounding fault detection. The MME coupling capability can be further enhanced by designing the rational spacing between the two arms in one U‐finger and adopting a symmetrical, variable stiffness structure.

Experimentally, the dual‐mode U‐finger A, an EH U‐finger, in the MME resonator could generate a high output power of 1.53 mW_RMS_ under a weak magnetic field of H_AC_ = 0.5 Oe at the resonance frequency of 50 Hz, and the MME coupling is 430% higher than that of single‐mode. At an extremely weak magnetic field of 0.1 Oe, this MME resonator also exhibits a 12.7% enhancement in normalized output power density compared with the state‐of‐art results. While the dual‐mode U‐finger B, a sensing U‐finger, can directly detect an extremely weak, only 710 pT, AC magnetic field at resonance frequency of 185 Hz, which is the given non‐grid‐frequency for grounding fault detection. The MME resonator was then integrated into a self‐powered non‐grid‐frequency current monitoring and communication system, demonstrating its capabilities in practical grid‐power line applications, including i) powering the signal reading module and the communication system, ii) sensing the non‐grid‐frequency injection current size via magnetoelectric coupling, and iii) transmitting the detected data to the data receiving terminal. The sustainability of the MME resonator and the ability to sense the state of the grid provide a viable solution for realizing the intelligence of future PIoT.

## 4 Experimental Section

### Fabrication of the MME Resonator

The substrate of the resonator is an elastic metal sheet made of 304 stainless steel with good mechanical properties and corrosion resistance. The piezoelectric material is the commercial supplied PZT‐5H ceramic sheets with a thickness of 0.2 mm (Pante, Shenzhen, China). This type of piezoelectric ceramic shows a high piezoelectric charge coefficient (see Table S1, Supporting Information), and its price is also much lower than that of piezoelectric single crystals.

First, the elastic metal sheet was laser cut into cross‐shaped beams and then folded into two U‐fingers using a bending process (as shown in Figure S1, Supporting Information). Then the piezoelectric ceramic sheets are bonded to each arm of the two U‐fingers with epoxy resin (105/206, West System, Bay City, MI, USA). After curing at room temperature for 24 h, the piezoelectric ceramic sheets were electrically connected in parallel with wires. The sizes of the longer U‐finger are 49 mm (L) × 20 mm (W) × 1.2 mm (T), and the sizes of the shorter U‐finger are 31 mm (L) × 20 mm (W) × 1.2 mm (T), respectively. The sizes of the piezoelectric ceramic sheet on the long arm are 35 mm (L) × 19 mm (W) × 0.2 mm (T), and the sizes of the piezoelectric sheet on the short arm are 18 mm (L) × 19 mm (W) × 0.2 mm (T). Finally, two pairs of permanent magnets (N52 NdFeB) were attached to the free end of each arm. There is a small hole in the center of the substrate plate for installation and fixing.

### Characterizations of the MME Resonator

A power amplifier (HA‐405, PINTEC) was used to power a pair of Helmholtz coils to generate an AC magnetic field. The double U‐finger MME resonator was placed at the center of the coils, and U‐finger A in the resonator was acted as MME energy harvester (MME‐EH) for harvesting magnetic field energy. A resistance box was used to provide the load resistance R_L_, and the output voltage V_RMS_ of the MME‐EH across the load resistance R_L_ can be measured through an oscilloscope (MSO‐5104, RIGOL Technologies, Suzhou New District, China). The output power *P* of the MME‐EH was calculated using $P\;=\frac{V_{\mathrm{RMS}}^{2}}{R_{L}}\;$.

A lock‐in amplifier (Malab, CIQTEK, Hefei, Anhui) was used to power the Helmholtz coil to generate an AC magnetic field with non‐grid‐frequency, and the ME voltage of the U‐finger B MME sensor was monitored by using an oscilloscope (MSOX4024A, Keysight). The ME voltage spectrum can be obtained by fast Fourier transform. In above experiments, two coaxial Helmholtz coils were used to generate the grid‐frequency and non‐grid‐frequency, respectively. The synchronous self‐powered and self‐sensing functions and output performances of the MME resonator were obtained by using the above‐mentioned experimental apparatus.

### Self‐Powered Non‐Grid‐Frequency Injection Current Monitoring and Communication System

50 and 185 Hz AC magnetic fields are generated by the power lines. The AC voltage generated by the EH U‐finger A in the MME resonator is converted into a DC impulse voltage through the piezoelectric energy management module (LTC3588, Kelan, Shenzhen, China) and a 1000 µF capacitor which then supplies power to the signal reading module (LRF 215, Kelan, Shenzhen, China). The ME voltage generated by the sensing U‐finger B in the MME resonator is converted into a DC voltage by a rectifier and stored in a 22 µF capacitor. At the same time, the signal reading module reads the 22 µF capacitor voltage, and finally sends the data to the data receiving terminal through the low‐power Zigbee communication module.

## Conflict of Interest

The authors declare no conflict of interest.

## Author Contributions

X.Z. and S.D. were responsible for conceptualization, methodology, investigation, visualization, and validation, and wrote the original draft. X.Z., B.W., W.P., Y.Y., H.P., Y.L., and X.L. conducted formal analysis. All authors participated in writing, reviewing, and editing the manuscript. S.D., J.C., S.F., and Z.C., performed project administration, acquired resources, and secured funding for the project.

## Supporting information



Supporting Information

## Data Availability

The data that support the findings of this study are available from the corresponding author upon reasonable request.
