# Supplementary material for: A Self‐Powered Double U‐Finger MME Resonator Capable of Wirelessly Capturing Abnormal Message in Smart Grid Networks
Source: Adv Sci (Weinh). 2025 Jul 11;12(36):e08149. doi: 10.1002/advs.202508149 (PMC12463033; doi:10.1002/advs.202508149)
Supplement: Supplementary file 1 — Supporting Information [file ADVS-12-e08149-s001.docx]

**A self-powered double U-finger MME resonator capable of wirelessly capturing abnormal message in Smart Grid Networks**

Xinyi Zheng^1^, Zhi Cheng^3^*, Bing Wang^1^, Wei Peng^6^, Yuelong Yu^1^, Haoxian Peng^1,5^, Yu Lei^7^, Xiangmeng Lv^1^, Jianglei Chang^4^*, Shitong Fang^1,5^*and Shuxiang Dong^2^*^,1^

^1^ School of Mechatronics and Control Engineering, Institute for Advanced Study, Shenzhen University, Guangdong 518060, China

^2^ School of Materials Science and Engineering, Peking University, Beijing 100871, China

^3^ School of Materials Science and Engineering, Wuhan University of Technology, Wuhan 430070, China

^4^ Electronic Materials Research Laboratory, Key Laboratory of the Ministry of Education, School of Electronic Science and Engineering, Xi’an Jiaotong University, Xi’an 710049, China

^5^ Guangdong Key Laboratory of Electromagnetic Control and Intelligent Robots, Shenzhen University, Guangdong 518060, China

^6^ Key Laboratory of Inorganic Functional Materials and Devices, Shanghai Institute of Ceramics, Chinese Academy of Sciences, Shanghai 201899, China

^7^ School of Aerospace Engineering, Beijing Institute of Technology, Beijing 100081, China

**Contents:**

**Section S1: The key geometric parameters of the double U-finger MME resonator.**

**Section S2: Equation of deflection curve of the single U-finger structure induced by magnetic torques.**

**Section S3: The power generation of the EH U-finger A.**

**Section S4: The sensing U-finger B response to step magnetic field.**

**Section S5: Linear relationship between power line current and ME voltage.**

**This file includes:**

Fig. S1 The geometric parameters of the double U-finger MME resonator.

Fig. S2 (a) Schematic view of the single U-finger structure. (b) Simplified mechanical model of half single U-finger structure. (c) Sectional views of different sections in the half single U-finger structure.

Fig. S3 Schematic diagram of magnetic field energy harvesting test system.

Fig. S4 The time-domain waveforms of open circuit voltage of the EH U-finger A under varying magnetic field intensities.

Fig. S5 The maximum average output power of the double U-finger MME resonator varies with the change of H_AC_.

Fig. S6 (a) The double U-finger MME resonator rotates about z-axis under H_AC_. (b) output power response when the double U-finger MME resonator rotates about the z-axis.

Fig. S7 Normalized peak-peak output voltages for lasting operations over 5.7 × 10^6^ cycles.

Fig. S8 Output ME voltage in response to an extremely weak step AC magnetic field variation.

Fig. S9 The sensing U-finger B has a linear response from 1 A to 3 A power line current.

Table S1. Basic piezoelectric parameters of the commercial PZT-5H ceramic.

**Section S1: The key geometric parameters of the double U-finger MME resonator.**


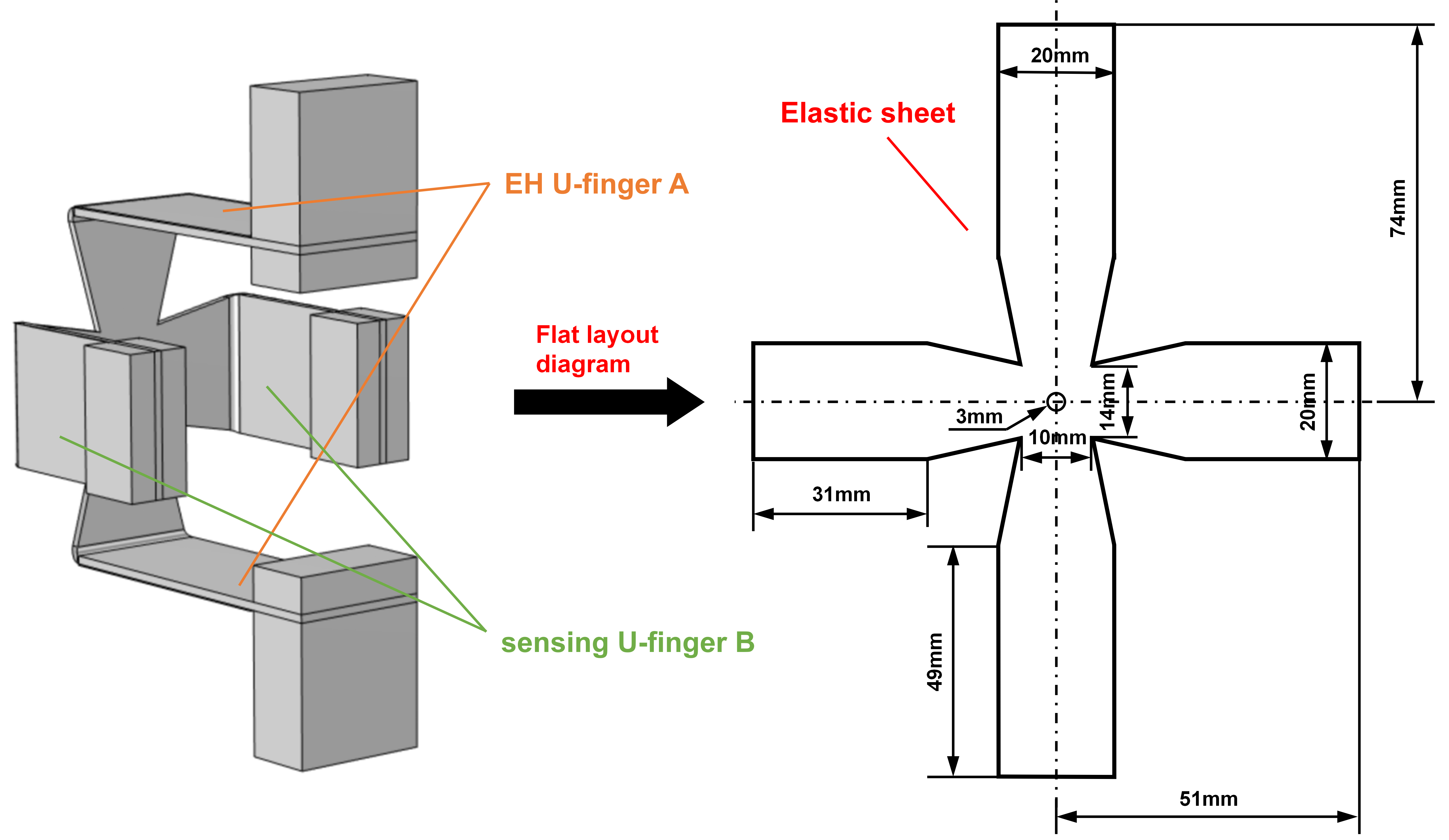


Fig. S1 The geometric parameters of the double U-finger MME resonator.

The elastic sheet, i.e., the substrate is made of 304 stainless steel with a thickness of 1.2 mm.

Table S1. Basic piezoelectric parameters of the commercial PZT-5H ceramic.

|  | d*_33_* | d*_15_* | g*_33_* | g*_15_* | *k_33_* | *Q_m_* | *tan*$\delta$ | *T_c_* |
| --- | --- | --- | --- | --- | --- | --- | --- | --- |
| PZT-5H | 593 | 741 | 19.7 | 26.8 | 0.75 | 65 | 2% | 193°C |

Inexpensive commercial piezoelectric ceramics possess relatively high piezoelectric coefficients and comparatively low losses, exhibiting excellent overall performance. This provides a guarantee for enhancing the output performance of double U-finger MME resonator.

**Section S2: Equation of deflection curve of the single U-finger structure induced by magnetic torques.**

**
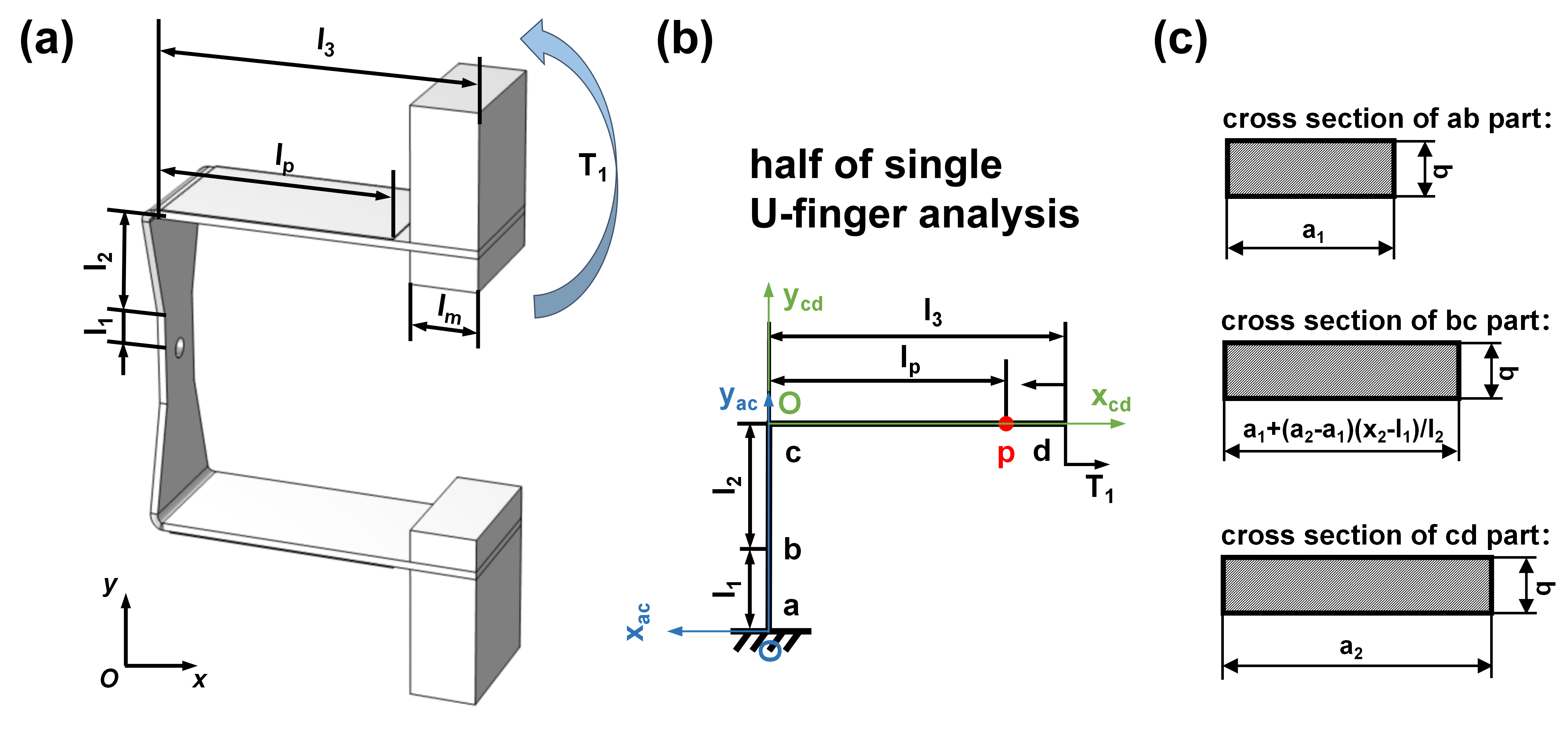
**

Fig. S2 (a) Schematic view of the single U-finger structure. (b) Simplified mechanical model of half single U-finger structure. (c) Sectional views of different sections in the half single U-finger structure.

In this work, the resonator is subjected to a bending moment M = T_1_ generated by H_AC_ induced magnetic torque. The displacement of the half U-finger is calculated using the displacement superposition method. First, by rigidifying the *cd* segment, we analyzed the *ac* segment. The bending moment equation in the *ac* segment can be written as

| $\left\{\begin{matrix} EI_{1}w''_{1}(y_{ac})=T_{1}\begin{matrix} & y_{ac}\in(0,l_{1}) \end{matrix} \\ EI_{2}w''_{2}(y_{ac})=T_{1}\begin{matrix} & y_{ac}\in(l_{1},l_{1}+l_{2}) \end{matrix} \end{matrix}\right.$ | (s1) |
| --- | --- |

Where $E$, $I$, $w''$ and are Yong's model of the elastic sheet (304 stainless steel), cross sectional moment of inertia, the second derivative of deflection and the segment length of the beam. The subscripts 1, 2 represent segments *ab* and *bc* of the beam, respectively. The expressions of *I_1_* and *I_2_* are:

| $\left\{\begin{matrix} I_{1}=\frac{a_{1}b^{3}}{12}\begin{matrix} & y_{ac}\in(0,l_{1}) \end{matrix} \\ I_{2}=\frac{(a_{2}-a_{1})(y_{ac}-l_{1})b^{3}+a_{1}b^{3}l_{2}}{12l_{2}}\begin{matrix} & y_{ac}\in(l_{1},l_{1}+l_{2}) \end{matrix} \end{matrix}\right.$ | (s2) |
| --- | --- |

Where *b* is the thickness of the beam. *a_1_* and *a_2_* are the widths of segments *ab* and *cd*, respectively. The rotation angle equations can be expressed as

| $\left\{\begin{matrix} w'_{1}(y_{ac})=\frac{T_{1}y_{ac}}{EI_{1}}+C_{1}\begin{matrix} & y_{ac}\in(0,l_{1}) \end{matrix} \\ w'_{2}(y_{ac})=\frac{C}{A}\ln(A(y_{ac}-l_{1})+B)+D_{1}\begin{matrix} & y_{ac}\in(l_{1},l_{1}+l_{2}) \end{matrix} \end{matrix}\right.$ | (s3) |
| --- | --- |

Where $A=E(a_{2}-a_{1})b^{3}$，$B=Ea_{1}b^{3}l_{2}$，$C=12T_{1}l_{2}$. , and are the rotation angle of *ab* segment and *bc* segment, respectively. The deflection equations are given by

| $\left\{\begin{matrix} w_{1}(y_{ac})=\frac{T_{1}y_{ac}^{2}}{2EI_{1}}+C_{1}y_{ac}+C_{2}\begin{matrix} & y_{ac}\in(0,l_{1}) \end{matrix} \\ w_{2}(y_{ac})=\frac{C}{A^{2}}((A(y_{ac}-l_{1})+B)\ln(A(y_{ac}-l_{1})+B)-(A(y_{ac}-l_{1})+B))+D_{1}y_{ac}+D_{2}\begin{matrix} & y_{ac}\in(l_{1},l_{1}+l_{2}) \end{matrix} \end{matrix}\right.$ | (s4) |
| --- | --- |

According to the boundary conditions and continuity conditions of *ab* and *bc* segments, we have

| $\left\{\begin{matrix} y_{ac}=0,w_{1}=0,w'_{1}=0 \\ y_{ac}=l_{1},w_{1}=w_{2}=w_{b},w'_{1}=w'_{2}=\theta_{b} \end{matrix}\right.$ | (s5) |
| --- | --- |

Where *w_b_* and *θ_b_* are the deflection and rotation angle of segment *ab* at point *b*, respectively. By substituting s5 into s3 and s4, the deflection expressions of two segments *ab* and *bc* can be obtained as follows:

| $\left\{\begin{matrix} w_{1}=\frac{6T_{1}y_{ac}^{2}}{Ea_{1}b^{3}}\begin{matrix} & y_{ac}\in(0,l_{1}) \end{matrix} \\ w_{2}=\frac{C}{A^{2}}((A(y_{ac}-l_{1})+B)\ln(A(y_{ac}-l_{1})+B)-(A(y_{ac}-l_{1})+B))+(\theta_{b}-\frac{C}{A}\ln B)(y_{ac}-l_{1})+w_{b}-\frac{C}{A^{2}}(B\ln B-B)\begin{matrix} & y_{ac}\in(l_{1},l_{1}+l_{2}) \end{matrix} \end{matrix}\right.$ | (s6) |
| --- | --- |

The rotation angle expressions can be obtained:

| $\left\{\begin{matrix} w'_{1}=\frac{12T_{1}y_{ac}}{Ea_{1}b^{3}}\begin{matrix} & y_{ac}\in(0,l_{1}) \end{matrix} \\ w'_{2}=\frac{C}{A}\ln(A(y_{ac}-l_{1})+B)+\theta_{b}-\frac{C}{A}\ln B\begin{matrix} & y_{ac}\in(l_{1},l_{1}+l_{2}) \end{matrix} \end{matrix}\right.$ | (s7) |
| --- | --- |

Therefore, the deflection and rotation angle of the segment *ac* at point *c* can be obtained:

| $\left\{\begin{matrix} w_{c}=\frac{C}{A^{2}}((Al_{2}+B)\ln(Al_{2}+B)-(Al_{2}+B))+(\theta_{b}-\frac{C}{A}\ln B)l_{2}+w_{b}-\frac{C}{A^{2}}(B\ln B-B) \\ w'_{c}=\theta_{c}=\frac{C}{A}\ln(Al_{2}+B)+\theta_{b}-\frac{C}{A}\ln B \end{matrix}\right.$ | (s8) |
| --- | --- |

Therefore, when rigidifing *cd* segment, the displacement of any point (*x_1_ , y_1_*) in the *cd* segment is:

| $\left\{\begin{matrix} x_{1}=w_{c}=\frac{C}{A^{2}}((Al_{2}+B)\ln(Al_{2}+B)-(Al_{2}+B))+(\theta_{b}-\frac{C}{A}\ln B)l_{2}+w_{b}-\frac{C}{A^{2}}(B\ln B-B) \\ y_{1}=\theta_{c}x=(\frac{C}{A}\ln(Al_{2}+B)+\theta_{b}-\frac{C}{A}\ln B)x \end{matrix}\right.$ | (s9) |
| --- | --- |

Next, by rigidifying the *ac* segment, we further analyzed the *cd* segment. The bending moment equation in the *cd* segment can be written as:

| $EI_{3}w''_{3}=T_{1}\begin{matrix} & x_{cd}\in(0,l_{3}) \end{matrix}$ | (s10) |
| --- | --- |

Where *I_3_*, *w՛՛_3_*, and *l_3_* are cross sectional moment of inertia, the second derivative of deflection and the length of *cd* segment. The expression of *I_3_* is

| $I_{3}=\frac{a_{2}b^{3}}{12}\begin{matrix} & x_{cd}\in(0,l_{3}) \end{matrix}$ | (s11) |
| --- | --- |

Similarly, the rotation angle and deflection of cd segment can be obtained as:

| $\left\{\begin{matrix} EI_{3}w'_{3}=T_{1}x_{cd}+P_{1}\begin{matrix} & x_{cd}\in(0,l_{3}) \end{matrix} \\ EI_{3}w_{3}=\frac{1}{2}T_{1}x_{cd}^{2}+P_{1}x_{cd}+P_{2}\begin{matrix} & x_{cd}\in(0,l_{3}) \end{matrix} \end{matrix}\right.$ | (s12) |
| --- | --- |

According to the boundary conditions of *cd* segment, we have

| $x_{cd}=0,w_{3}=0,w'_{3}=0$ | (s13) |
| --- | --- |

After substituting s13 into s12, the expressions of rotation angle and deflection of *cd* segment are given as

| $\left\{\begin{matrix} w_{3}=\frac{6T_{1}x_{cd}^{2}}{Ea_{2}b^{3}}\begin{matrix} & x_{cd}\in(0,l_{3}) \end{matrix} \\ w'_{3}=\frac{12T_{1}x_{cd}}{Ea_{2}b^{3}}\begin{matrix} & x_{cd}\in(0,l_{3}) \end{matrix} \end{matrix}\right.$ | (s14) |
| --- | --- |

Therefore, when rigidifing *ac* segment, the displacement of any point (*x_2_ , y_2_*) in the *cd* segment is:

| $\left\{\begin{matrix} x_{2}=0 \\ y_{2}=\frac{6T_{1}x^{2}}{Ea_{2}b^{3}} \end{matrix}\right.$ | (s15) |
| --- | --- |

Thus, the horizontal displacement and vertical displacement of each point in the cd segment are:

| $\left\{\begin{matrix} x=x_{1}+x_{2}=\frac{C}{A^{2}}((Al_{2}+B)\ln(Al_{2}+B)-(Al_{2}+B))+(\theta_{b}-\frac{C}{A}\ln B)l_{2}+w_{b}-\frac{C}{A^{2}}(B\ln B-B) \\ y=y_{1}+y_{2}=(\frac{C}{A}\ln(Al_{2}+B)+\theta_{b}-\frac{C}{A}\ln B)x+\frac{6T_{1}x^{2}}{Ea_{2}b^{3}} \end{matrix}\right.$ | (s16) |
| --- | --- |

The length of the fixed piezoelectric piece is $l_{p}=35mm$, and the vertical displacement of the end of the piezoelectric piece can be expressed as:

| $y_{p}=y(l_{p})=(\frac{C}{A}\ln(Al_{2}+B)+\theta_{b}-\frac{C}{A}\ln B)l_{p}+\frac{6T_{1}l_{p}^{2}}{Ea_{2}b^{3}}$ | (s13) |
| --- | --- |

**Section S3: The power generation of the EH U-finger A.**


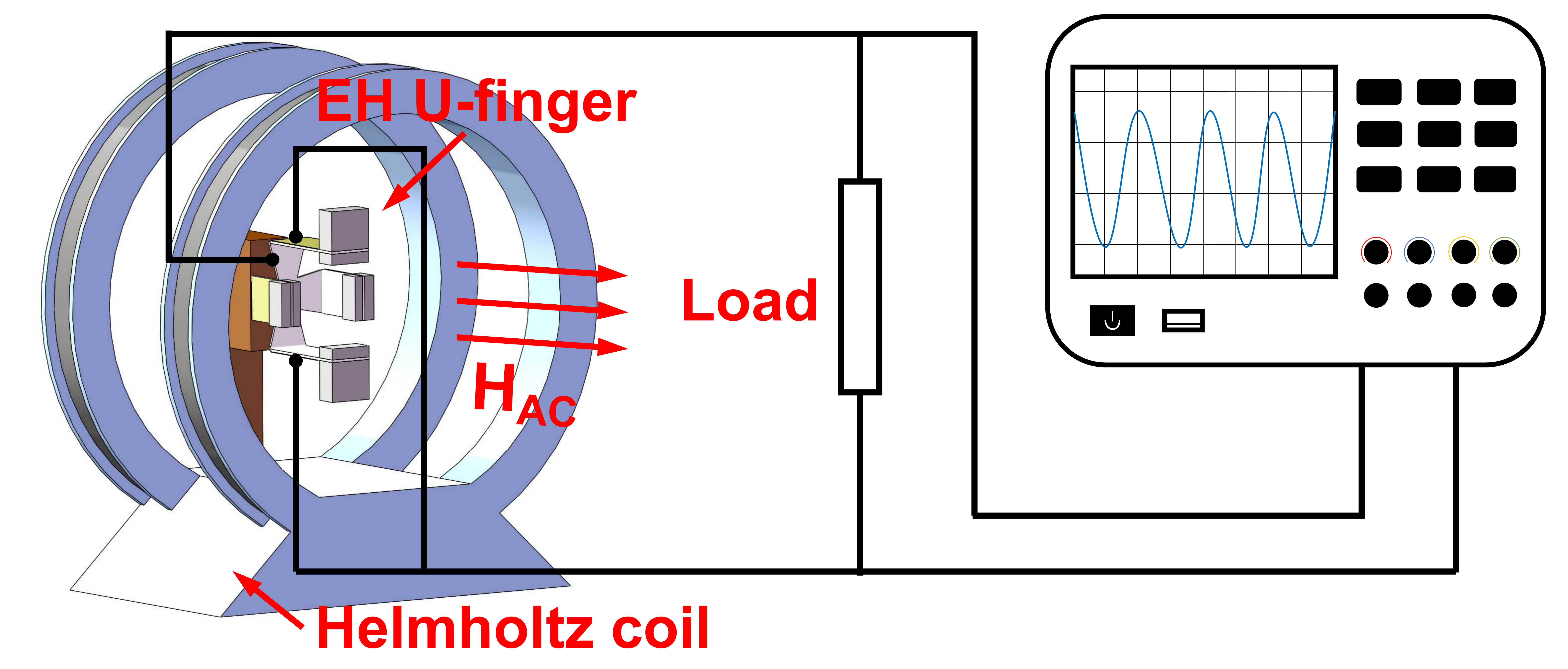


Fig. S3 Schematic diagram of magnetic field energy harvesting test system.


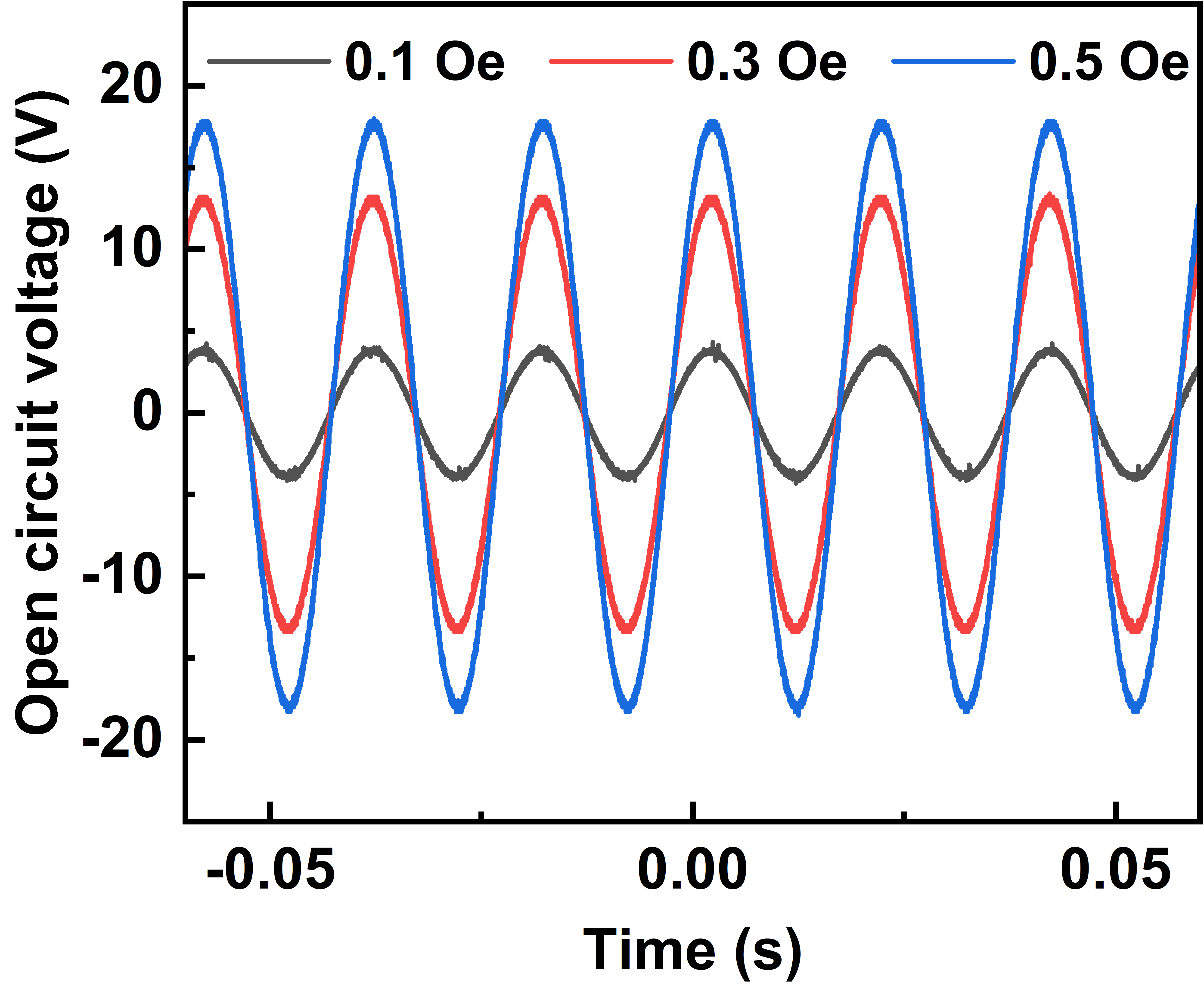


Fig. S4 The time-domain waveforms of open circuit voltage of the EH U-finger A under varying magnetic field intensities.


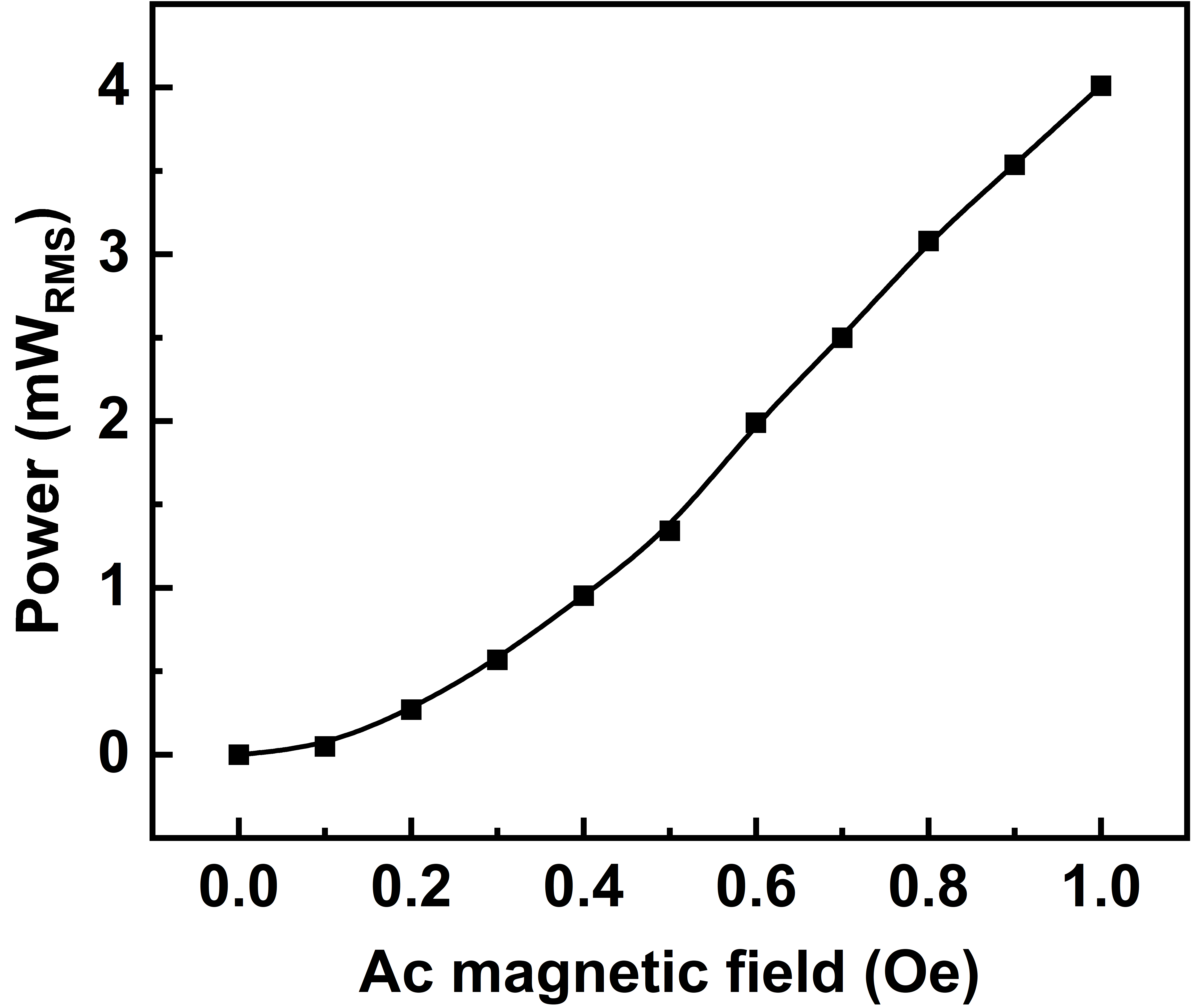


Fig. S5 The maximum average output power of the double U-finger MME resonator varies with the change of H_AC_.


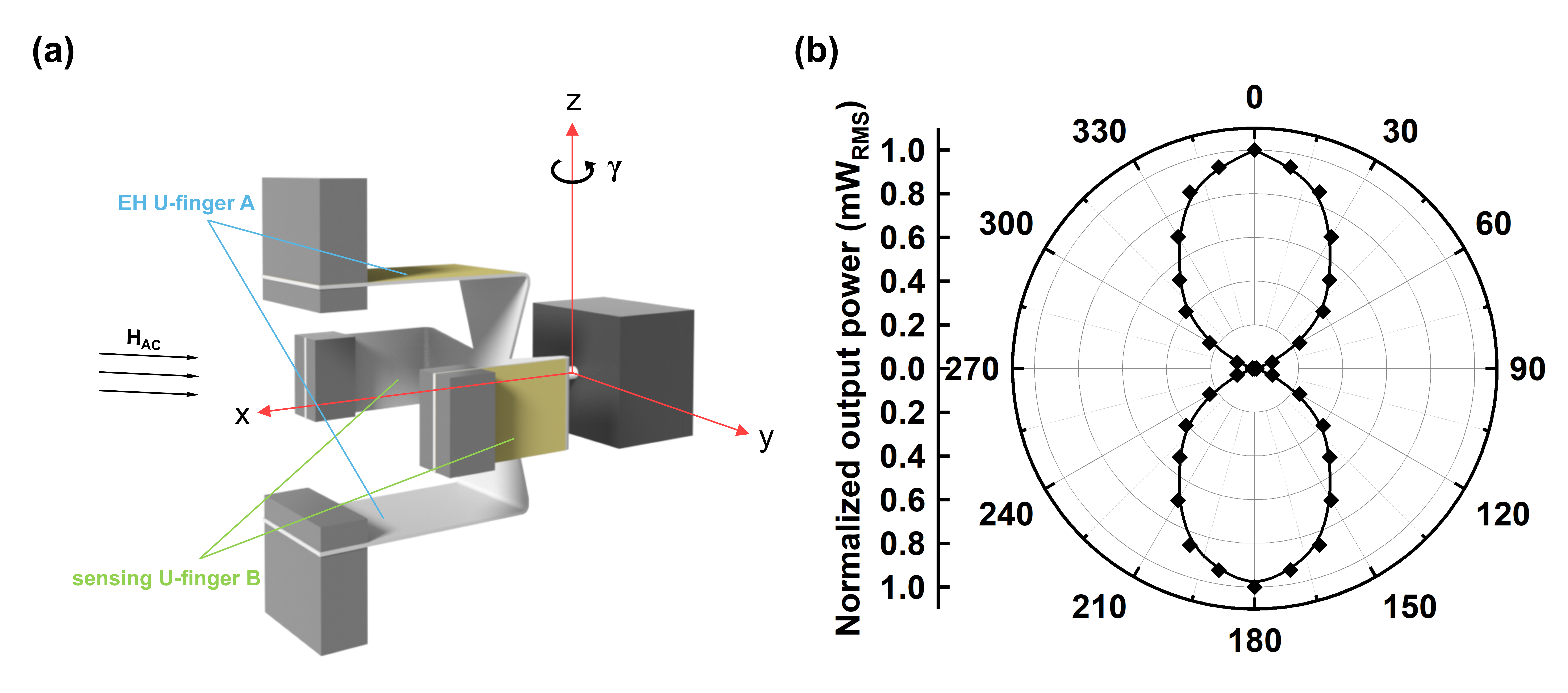


Fig. S6 (a) The double U-finger MME resonator rotates about z-axis under H_AC_. (b) output power response when the double U-finger MME resonator rotates about the z-axis.


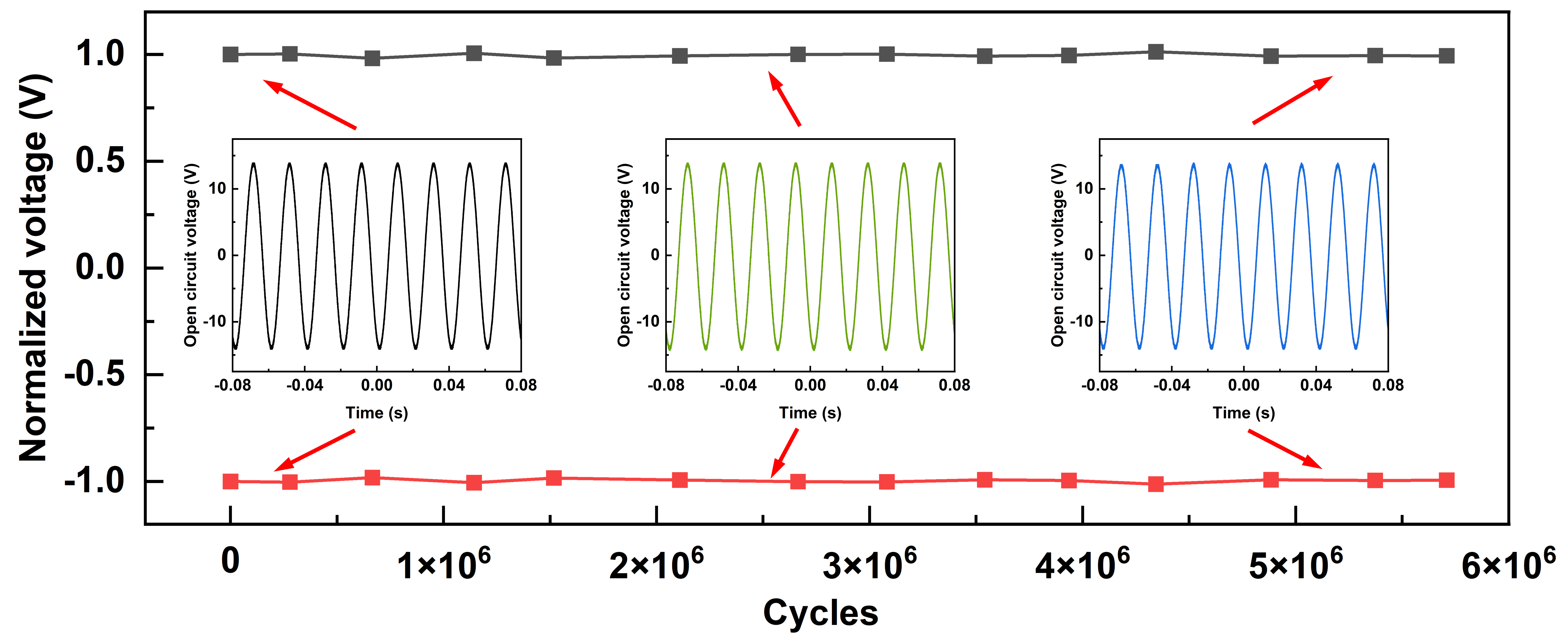


Fig. S7 Normalized peak-peak output voltages for lasting operations over 5.7 $\times$ 10^6^ cycles.

Fig. S7 shows that the EH U-finger A still exhibits a stable voltage response after lasting operations over 5.7 $\times$ 10^6^ cycles under the excitation of an AC magnetic field of 0.3 Oe with the frequency of 50 Hz. This fatigue test validates the reliability of the EH U-finger A under long-term operation.

**Section S4: The sensing U-finger B response to step magnetic field.**

**
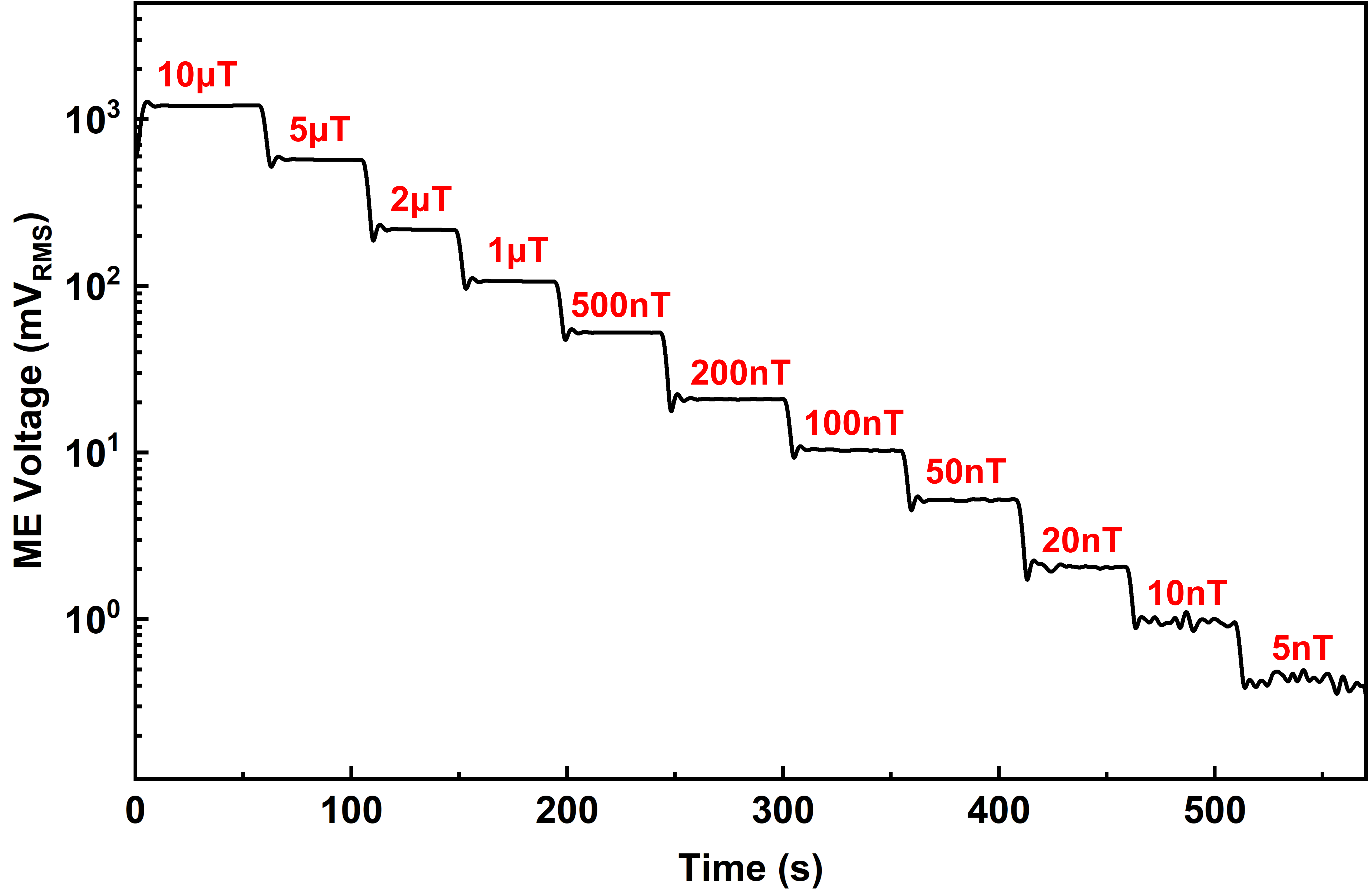
**

Fig. S8 Output ME voltage in response to an extremely weak step AC magnetic field variation.

**Section S5: Linear relationship between power line current and ME voltage.**


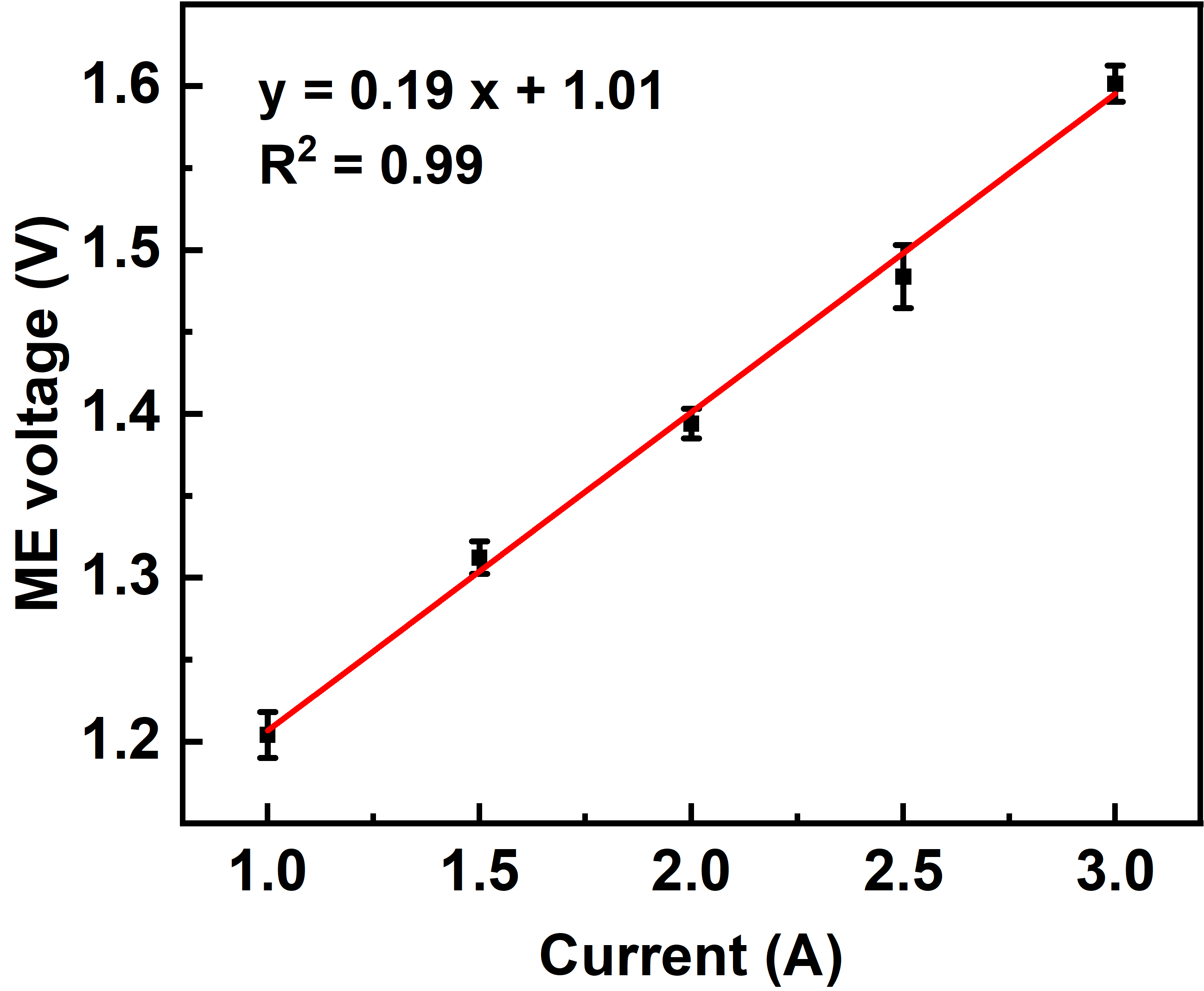


Fig. S9 The sensing U-finger B has a linear response from 1 A to 3 A power line current.
